# Case Report: Bezoar-mimicking intraluminal gossypiboma causing small-bowel obstruction and chronic malabsorption 15 years after hysterectomy

**DOI:** 10.3389/fmed.2026.1930006

**Published:** 2026-08-26

**Authors:** Marcel Antonio Cazarez-Aguilar, Francisco Magaña-Olivas, Martín Adrián Bolívar-Rodríguez, Pedro Alejandro Magaña-Zavala, Miguel Abelardo Rentería-Solís, Jorge Angulo-Rocha, Nidia León-Sicairos, Adrian Canizalez-Roman, Jesús Javier Martínez-García

**Affiliations:** 1 Centro de Investigación y Docencia en Ciencias de la Salud (CIDOCS), Cirugía General, Hospital Civil de Culiacán, Culiacán, Sinaloa, Mexico; 2 School of Medicine, Autonomous University of Sinaloa, Culiacan, Sinaloa, Mexico; 3 Coordinación de Quirófano, Hospital Médica de la Ciudad, Culiacán, Sinaloa, Mexico; 4 Research Department, The Women’s Hospital, Secretariat of Health, Culiacán, Sinaloa, Mexico; 5 Research Department, Pediatric Hospital of Sinaloa, Culiacán, Sinaloa, Mexico

**Keywords:** computed tomography, gossypiboma, retained surgical sponge, small-bowel obstruction, textiloma, transmural migration

## Abstract

**Background:**

Gossypiboma (textiloma) denotes a retained surgical textile enclosed by a foreign-body reaction. It may remain silent for years or present with nonspecific symptoms; transmural migration into the bowel lumen is a rare complication that can cause obstruction or malabsorption and is frequently mistaken for other intraluminal masses.

**Case presentation:**

A 65-year-old woman with a total abdominal hysterectomy for uterine myomatosis 15 years earlier presented with 15 days of right-upper-quadrant pain and gastrobiliary vomiting, superimposed on 3 years of intermittent chronic diarrhea and a 15-kg weight loss in the preceding year. She was hypotensive and dehydrated, with hyponatremia and acute kidney injury. Unenhanced multiplanar abdominopelvic CT, intravenous contrast having been withheld because of the acute kidney injury and enteral contrast because of vomiting and obstruction, showed an intraluminal right-flank mass with a mottled, spongiform pattern containing gas, mural thickening with mesenteric fat stranding and laminar free fluid, a transition point with proximal small-bowel dilatation and air–fluid levels, and retrograde dilatation of the proximal loops. No radio-opaque marker was identified, and the mass was initially interpreted as a bezoar. Because she was managed emergently, no dedicated malabsorption workup was performed, and the malabsorptive syndrome remained a clinical diagnosis. At emergency laparotomy, a paracecal internal hernia and a bezoar-like intraluminal mass 30 cm from the ileocecal valve were found; *en bloc* resection of the affected ileum and sigmoid colon with a diverting ileostomy and mucous fistula was performed. Histopathology confirmed a 30-cm surgical gauze. She recovered uneventfully and was discharged on postoperative day 5.

**Conclusion:**

Intraluminal gossypiboma should be considered in any patient with previous abdominal or pelvic surgery who presents with chronic refractory diarrhea, malabsorption, or unexplained bowel obstruction, even decades later. CT is the modality of choice, although its diagnostic yield falls when intravenous contrast cannot be administered and no radio-opaque marker is visible, as occurred here; definitive management is surgical removal. Follow-up was limited to the early postoperative period; stoma reversal and confirmation of resolution of the malabsorptive syndrome remain pending. Rigorous surgical counts and radio-opaque-marked textiles remain the cornerstone of prevention.

## 1 Introduction

Gossypiboma, from the Latin gossypium (cotton) and the Swahili boma (place of concealment) describes a mass formed around a textile foreign body (cotton sponge, gauze, dressing, or laparotomy pad) inadvertently left in the body after surgery; the terms textiloma, cottonoid, and gauzeoma are used synonymously (1). It complicates abdominal, thoracic, cardiovascular, orthopedic, and neurosurgical procedures, with a reported incidence of roughly 1 per 1,000–1,500 intra-abdominal operations, although the true frequency is uncertain because many cases are asymptomatic and underreported for medico-legal reasons (2).

Retained textiles elicit one of two foreign-body responses: an exudative/septic reaction producing an abscess or fistula, or an aseptic fibrotic reaction that encapsulates the material and favors detection on computed tomography (CT) (3). Recognized complications include adhesions, fistulae, abscesses, gastrointestinal ulceration, hemorrhage from vessel erosion, and rarely transmural migration into an adjacent hollow viscus (3, 4). Intraluminal migration into the small bowel is exceptionally uncommon; when it occurs, the ileocecal valve is the most frequent site of obstruction, and presentations range from acute obstruction to chronic malabsorption (4).

Two features make the present case instructive. First, the retained gauze mimicked a bezoar both clinically and radiologically, a preoperative misinterpretation seldom reported, since previously described mimics are more often gastrointestinal stromal tumors, calcified parasites, acute appendicitis, or abscessed colonic tumors (5). Second, migration presented after an unusually long 15-year latency and was dominated by chronic, refractory diarrhea and malabsorption rather than acute obstruction, a phenotype that is underrepresented in the literature (6). The case additionally illustrates the limits of cross-sectional imaging when a retained textile must be characterized without intravenous contrast and in the absence of a detectable radio-opaque marker. We report this case to highlight these diagnostic pitfalls and to reinforce prevention.

## 2 Case description

### 2.1 Patient information and history

A 65-year-old woman presented to the emergency department with right-upper-quadrant abdominal pain and vomiting of gastrobiliary content of 15 days’ duration, which had increased in intensity and frequency over the preceding 3 days. Her medical history included systemic arterial hypertension, type 2 diabetes mellitus, and hypercholesterolemia of approximately 10 years’ evolution. Fifteen years earlier, she had undergone a total abdominal hysterectomy for uterine myomatosis. Over the preceding 3 years, she had experienced intermittent episodes of chronic diarrhea refractory to medical treatment, with a 15-kg weight loss during the last year. She reported no relevant family or psychosocial history. A timeline of the episode of care is summarized in Table 1.

**Table 1:** Timeline of the episode of care.

Time (relative to admission)	Event
15 years before	Total abdominal hysterectomy for uterine myomatosis (index operation).
Preceding 3 years	Intermittent episodes of chronic diarrhea, refractory to medical treatment.
Preceding 12 months	Progressive weight loss of ∼15 kg.
15 days before	Right-upper-quadrant abdominal pain and gastrobiliary vomiting.
Last 3 days	Worsening intensity and frequency of pain and vomiting.
Day 0 (admission)	Hypotension, dehydration, hyponatremia, and acute kidney injury; unenhanced multiplanar abdominopelvic CT showed an intraluminal foreign body with a transition point and proximal dilatation; nasogastric decompression and fluid resuscitation started.
Day 0–1	Emergency exploratory laparotomy: paracecal internal hernia and intraluminal bezoar-like mass; *en bloc* ileal–sigmoid resection with diverting ileostomy and mucous fistula.
Postoperative	Histopathology confirmed a 30-cm surgical gauze (gossypiboma).
Day 5	Discharge after uneventful recovery, adequate oral tolerance, and normal stoma function; no follow-up cross-sectional imaging was obtained.

### 2.2 Clinical findings

On examination, she was in poor general condition, pale, with dehydrated oral mucosa. Blood pressure was 83/36 mmHg, heart rate 76 beats/min, and temperature 36.2 °C. The abdomen showed reduced peristalsis, tenderness in all quadrants, tympany, and a positive Blumberg (rebound) sign. Laboratory studies revealed hyponatremia (sodium 130.6 mmol/L), hyperglycemia (glucose 236 mg/dL), and elevated nitrogenous products (urea 158 mg/dL; creatinine 1.85 mg/dL), consistent with hypovolemic shock and acute kidney injury.

### 2.3 Diagnostic assessment

**Figure 1:**
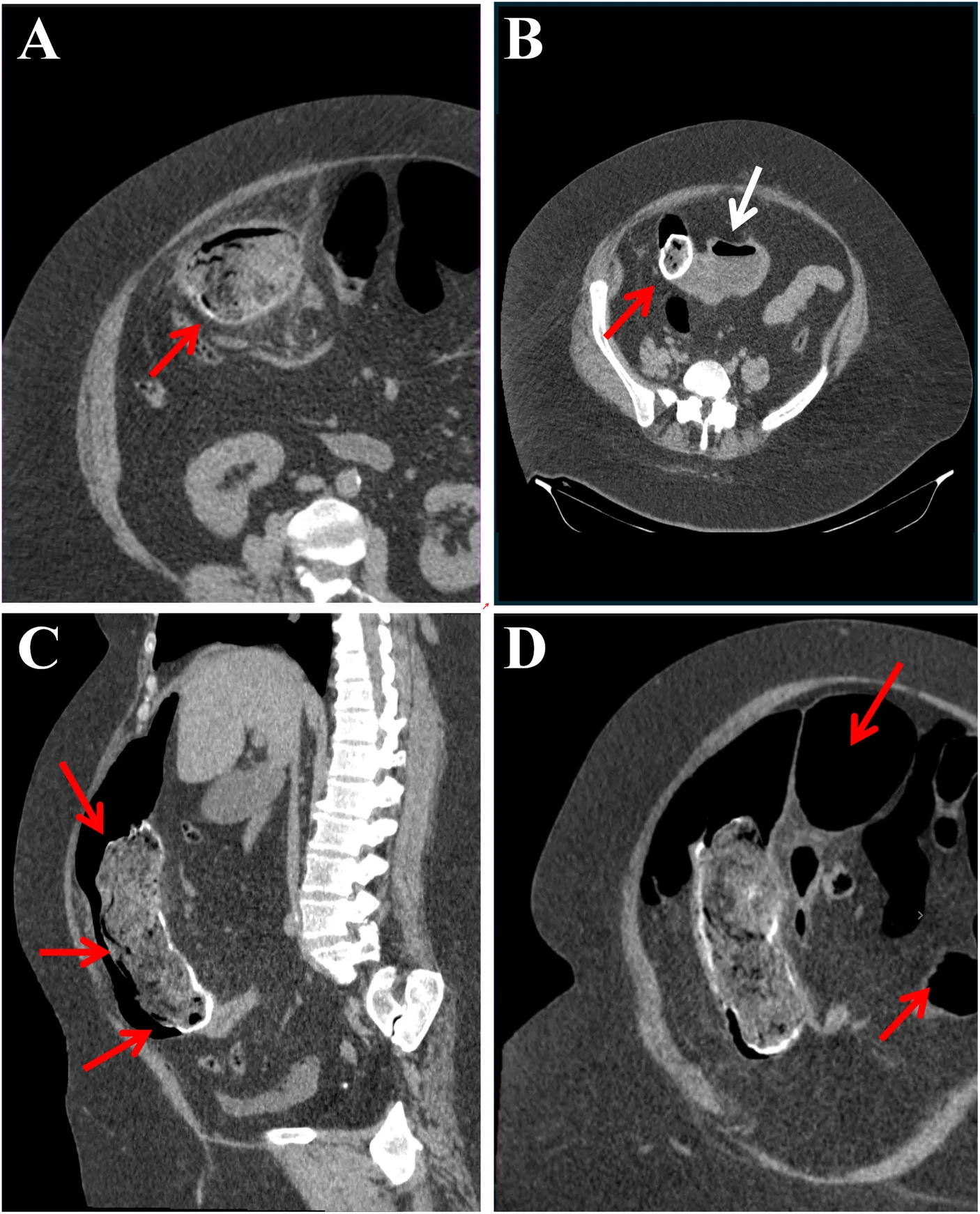
Unenhanced multiplanar abdominopelvic CT at admission. The study was acquired in the unenhanced phase: intravenous iodinated contrast was withheld because of the acute kidney injury present on admission in a hypotensive, hypovolaemic patient, and enteral contrast was not administered because of persistent vomiting and clinical obstruction. **(A)** Axial image at the level of the lesion showing the intraluminal mass with a mottled, spongiform internal pattern containing entrapped gas (red arrow); the wall of the affected ileal loop is thickened, and the adjacent mesenteric fat shows stranding with laminar free fluid, indicating compromise of the bowel wall. **(B)** Axial image acquired caudal to the mass, demonstrating the obstructive intraluminal lesion at the transition point (red arrow), with proximal small-bowel dilatation and an air–fluid level (white arrow). **(C)** Oblique sagittal reformat showing the craniocaudal extent of the intraluminal textiloma in the right flank (red arrows), with hyperdense material along its postero-inferior margin and edema of the adjacent mesenteric fat. **(D)** Oblique coronal reformat demonstrating retrograde dilatation of the proximal small bowel with abundant intraluminal gas (red arrows). No radio-opaque marker attributable to a surgical textile was identified on any plane. Because the examination was unenhanced, capsular enhancement and mural perfusion could not be evaluated.

Imaging: Multiplanar abdominopelvic CT was obtained without contrast. Intravenous iodinated contrast was withheld because of the acute kidney injury documented on admission (creatinine 1.85 mg/dL, urea 158 mg/dL) in a patient who was simultaneously hypotensive and hypovolaemic; the decision was individualized in line with consensus recommendations on the use of iodinated contrast media in patients with reduced renal function (7). Enteral contrast was likewise not administered, because of persistent vomiting and clinically evident obstruction, a setting in which positive luminal contrast adds little diagnostically and carries a risk of aspiration, and in which the fluid-filled dilated loops provide intrinsic contrast (18). An intraluminal mass was identified in the right flank, displaying a mottled, spongiform internal pattern with entrapped gas and hyperdense material along its postero-inferior margin (Figures 1A,C). The affected ileal loop showed mural thickening, mesenteric fat stranding and adjacent laminar free fluid; these findings indicate compromise of the bowel wall, although mural perfusion could not be assessed in the absence of intravenous contrast (Figure 1A). A transition point was demonstrated caudal to the mass, with proximal small-bowel dilatation and an air–fluid level (Figure 1B), and oblique coronal reformats confirmed retrograde dilatation of the proximal loops with abundant gas (Figure 1D). No radio-opaque marker attributable to a surgical textile was identified on any plane.

The preoperative differential diagnosis included a bezoar, an intraluminal tumor, and a retained foreign body; given the chronic malabsorptive history and dietary factors, a bezoar was considered the leading preoperative diagnosis. Two technical circumstances contributed to this misinterpretation: the absence of intravenous contrast, which precludes demonstration of the peripheral capsular enhancement that favors a retained textile, and the absence of a visible radio-opaque marker, which removes the single most specific imaging sign of gossypiboma.

Studies of intestinal absorption: No dedicated malabsorption workup quantitative or qualitative fecal fat, D-xylose absorption testing, fecal elastase, breath testing for small-intestinal bacterial overgrowth, celiac serology, or a formal nutritional and micronutrient panel was performed, because the patient was admitted with an acute abdomen, hypovolemic shock, and acute kidney injury and underwent emergency surgery within the first 24 h; no records of previous outpatient investigations for her chronic diarrhea were available. The malabsorptive syndrome therefore remained a clinical diagnosis, based on the 3-year history of chronic diarrhea refractory to medical treatment and the 15-kg weight loss, together with the admission laboratory abnormalities described above.

### 2.4 Therapeutic intervention

**Figure 2:**
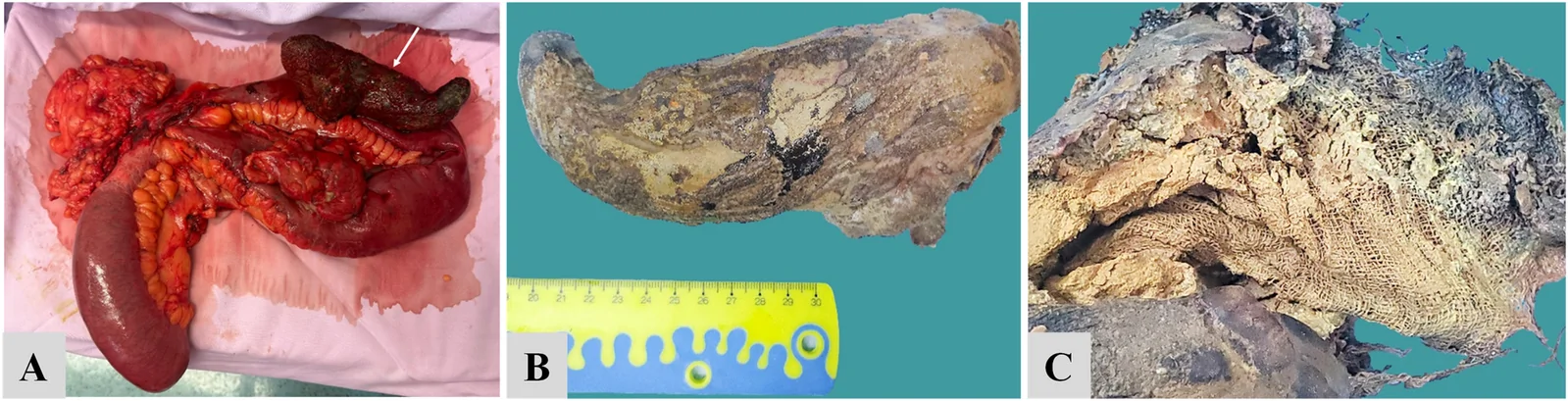
Operative and pathological findings. **(A)** Macroscopic appearance of the *en bloc* surgical specimen after resection, showing the congested and partially necrotic ileal segment; the white arrow indicates the dilated portion containing the intraluminal gossypiboma. **(B)** Encapsulated gossypiboma after removal from the bowel lumen; the ruler provides scale. **(C)** A 30-cm surgical gauze retrieved after incision of the capsule and shown unfolded; note the woven texture of the textile and the absence of a visible radio-opaque filament.

Initial resuscitation comprised nasogastric decompression and 0.9% saline boluses to correct hypovolemic shock and hyponatremia. An emergency exploratory laparotomy was performed. A paracecal internal hernia over the mesentery was found, incarcerating approximately 1 m of ileum together with part of the sigmoid colon. Dense adhesions were present in the pericecal region and around the affected loop, in continuity with the field of the previous operation; the hernia was therefore not interpreted as an isolated incidental finding (see Discussion). After reduction of the hernia, a firm, bezoar-like intraluminal mass was palpated 30 cm from the ileocecal valve. The ileum and sigmoid colon were densely adherent and showed necrotic tissue, corresponding to the mural thickening, fat stranding, and laminar free fluid observed preoperatively on CT (Figure 1A). An *en bloc* resection of ∼1 m of ileum and the involved sigmoid colon was carried out with a mechanical stapler, and a functional (diverting) ileostomy with a descending-colon mucous fistula was fashioned (Figure 2A).

### 2.5 Follow-up and outcomes

Histopathological examination of the intraluminal mass described a fecaloid-appearing specimen measuring 14 × 7 cm, fetid, with a granular surface, corresponding to a 30-cm surgical gauze embedded with fecal matter, confirming a gossypiboma (Figures 2B,C). The gauze was folded upon itself within the specimen, which accounts for the discrepancy between the textile's unfolded length (30 cm) and the dimensions of the retrieved mass. No radio-opaque filament was identified within the retrieved textile on macroscopic inspection, in keeping with the absence of such a marker on CT. The postoperative course was uneventful, with adequate oral tolerance and normal stoma function; the patient was discharged on postoperative day 5. No postoperative or follow-up cross-sectional imaging was obtained: the postoperative course was uncomplicated, and imaging was not clinically indicated before discharge, so CT images at the time of clinical re-evaluation are not available for this report. Follow-up was limited to the early postoperative period; longer-term outcomes, including planned stoma reversal and objective documentation of resolution of the malabsorptive syndrome, were not available at the time of reporting.

## 3 Discussion

We present a gossypiboma caused by surgical gauze retained after a hysterectomy performed 15 years earlier. Transmural migration of an intra-abdominal gossypiboma into the small bowel is a rare and serious complication (6, 8). The retained textile provokes either an infectious/exudative reaction, which may form an abscess or entero-cutaneous fistula and can facilitate earlier diagnosis, or a non-infectious fibrotic reaction that encapsulates the material and is more readily identified on CT (3). The prevailing hypothesis for luminal migration is that the exudative inflammatory response gradually erodes the bowel wall, allowing the foreign body to be extruded into the lumen, sometimes without leaving a persistent defect; the ileocecal valve is the commonest site of resulting obstruction (4, 8).

Clinical manifestations of small-bowel gossypiboma include abdominal pain, nausea, vomiting, anemia, a palpable mass, diarrhea, malabsorption secondary to enteric fistulae or intraluminal bacterial overgrowth, and weight loss (9). Contrast-enhanced CT is the diagnostic modality of choice; the characteristic finding is a well-defined encapsulated mass with a central spongiform pattern containing gas bubbles, although these features are suggestive rather than pathognomonic and diagnostic accuracy declines appreciably under the conditions discussed below (10). In the present patient, the examination was necessarily unenhanced, so this discriminator was unavailable. In our patient, these features were present but were interpreted preoperatively as a bezoar because of the chronic malabsorptive history and dietary factors, frequent consumption of fibrous fruits (mango, guava, strawberry), and impaired mastication from missing dentition, which are recognized predisposing conditions for bezoar formation (11, 12).

Imaging features and diagnostic pitfalls: review of the evidence. The CT appearance of a retained textile has been characterized in dedicated imaging reviews (13, 14). The most consistent finding is a well-circumscribed mass with a whorled or spongiform internal architecture produced by gas trapped within the interstices of the cotton mesh; this gas is a property of the textile itself and does not, in isolation, indicate superimposed infection (13). A peripheral capsule that enhances after intravenous contrast reflects the fibro-inflammatory reaction and is a helpful discriminator from luminal contents, but it can only be assessed on a contrast-enhanced study (13, 14). The radio-opaque marker, when present and intact, is the single most specific sign; it may nevertheless be undetectable when the textile predates the routine use of marked materials, when the marker has fragmented or degraded over prolonged retention, or when it is obscured by dense fecal or mineralized material within the bowel lumen (14). Differentiating a migrated textile from a phytobezoar is particularly difficult, since both may appear as a mottled intraluminal mass with entrapped gas at a transition point; reported series indicate that the distinction is frequently made only at operation (2, 14). The present case reproduced several of these limitations simultaneously: an unenhanced study mandated by acute kidney injury, no identifiable radio-opaque marker, and an intraluminal location within a feces-filled ileum, which together account for the preoperative attribution to a bezoar. This experience supports a practical recommendation: in a patient with prior abdominal or pelvic surgery and an intraluminal mass of spongiform appearance, a retained textile should be considered even when the marker is not visible and even when contrast cannot be administered, and the operative team should be alerted to the possibility before laparotomy.

This case adds to the small body of literature in three respects. First, documented preoperative misdiagnosis as a bezoar is rare; reported radiological mimics more often include a gastrointestinal stromal tumor, a calcified parasite, acute appendicitis, and an abscessed colonic tumor (5), and patients may even be told a mass is potentially malignant, prompting unnecessary extirpative surgery. Second, the 15-year latency and a clinical picture dominated by chronic, refractory diarrhea and malabsorption rather than acute obstruction represent an underreported phenotype, since most migratory gossypibomas present acutely or subacutely (6, 8). Third, the coexistence of an internal paracecal hernia with the intraluminal gossypiboma is an unusual combination that compounded the obstructive presentation. Together, these features underscore that intraluminal gossypiboma belongs in the differential diagnosis of chronic post-surgical malabsorption and unexplained bowel obstruction, even decades after the index operation.

Relationship between the internal hernia and the gossypiboma. Pericecal hernias usually occur through pre-existing congenital peritoneal recesses around the cecum, the superior and inferior ileocecal, retrocecal, and paracecal fossae, rather than through defects created *de novo* (15). In the present case, however, we do not regard the hernia as a purely coincidental finding. The patient had a dense adhesive syndrome attributable to the index hysterectomy and, superimposed on it, a chronic peritoneal foreign-body inflammatory reaction induced by the retained gauze. We propose that these two processes fixed and angulated the ileal loops and thereby favored their entrapment and later incarceration within a pericecal fossa, while the intraluminal gauze itself acted as an obstructive point 30 cm from the ileocecal valve, so that the two lesions compounded one another. This mechanism is biologically plausible but cannot be proven in a single case; a contributory rather than a strictly causal role is the most defensible interpretation, and at minimum both findings should be regarded as pathophysiologically linked through the same previous operation.

Comparable reports support this teaching. Kato et al. described a 57-year-old woman with intestinal obstruction from complete transmural sponge migration 18 years after hysterectomy (16); exploratory laparotomy revealed dense adhesions 60 cm from the terminal ileum, two fistulae communicating with the proximal jejunum, and a hard small-bowel mass that proved to be a surgical gauze, with granulation tissue around the foreign material on pathology. In our patient, who presented 15 years after the index operation, a 3-year history of intermittent chronic diarrhea and malabsorption unresponsive to medical therapy preceded the acute obstructive presentation.

Strengths and limitations. The strengths of this report include histopathological confirmation and complete imaging–surgical correlation. Its limitations are those inherent to a single case and a short follow-up that does not capture long-term outcomes such as stoma reversal or resolution of the malabsorptive syndrome. In addition, no dedicated malabsorption testing (fecal fat, D-xylose, fecal elastase, breath tests, celiac serology, or micronutrient panels) was performed because the patient was managed emergently; the malabsorptive syndrome is therefore clinically presumed rather than biochemically documented, and its causal attribution to the intraluminal gauze remains inferential. Two further limitations concern imaging. First, the examination was acquired in the unenhanced phase because intravenous iodinated contrast was withheld due to acute kidney injury. We acknowledge that contrast-enhanced CT is the preferred technique when small-bowel obstruction with possible strangulation is suspected (18), and that the unenhanced acquisition was therefore a compromise imposed by the renal status of the patient rather than a preferred strategy. Capsular enhancement, one of the discriminators between a retained textile and other intraluminal contents, could not be evaluated, and neither could mural perfusion, so the extent of bowel-wall compromise was inferred from wall thickening, mesenteric fat stranding and laminar free fluid rather than demonstrated directly. Second, no postoperative or follow-up cross-sectional imaging was obtained, since the uncomplicated postoperative course provided no indication for it; radiological documentation of the resolved obstruction at re-evaluation is consequently not available. Finally, the short follow-up does not capture long-term outcomes such as stoma reversal or objective resolution of the malabsorptive syndrome, which would have provided retrospective confirmation of that causal link.

## 4 Conclusion

A gossypiboma should be considered in the differential diagnosis of any patient with prior abdominal or pelvic surgery who presents with intestinal obstruction or a malabsorptive syndrome refractory to treatment, even many years after the operation. The diagnosis should be entertained even when CT cannot be performed with intravenous contrast and when no radio-opaque marker is identified, since neither circumstance excludes a retained textile. In patients presenting with a malabsorptive syndrome, the causal role of the retained textile can only be confirmed retrospectively, through resolution of the syndrome after its surgical removal; in the present case, both stoma reversal and documentation of such resolution remain pending and were not available at the time of reporting, and our conclusions should be interpreted with this in mind. Prevention rests primarily on meticulous instrument and sponge counts before and after surgery and on the routine use of radio-opaque-marked textiles, bearing in mind that these markers are inert radiographic filaments and not radioactive labels, and that counts documented as correct do not exclude a retained item.

## 5 Patient perspective

For years after my hysterectomy, I lived with abdominal pain and diarrhea that no treatment relieved, and I lost a great deal of weight. Learning that the cause was a surgical gauze left from that earlier operation was distressing, but I am relieved that the problem was finally identified and treated and that I have recovered well.

## Data Availability

The original contributions presented in the study are included in the article, further inquiries can be directed to the corresponding author.
